# Stability Analysis for Memristor-Based Complex-Valued Neural Networks with Time Delays

**DOI:** 10.3390/e21020120

**Published:** 2019-01-28

**Authors:** Ping Hou, Jun Hu, Jie Gao, Peican Zhu

**Affiliations:** 1 School of Management, Nanjing University of Posts and Telecommunications, Nanjing 210023, China; 2 School of Management Science and Engineering, Central University of Finance and Economics, Beijing 100080, China; 3 School of Sciences, Southwest Petroleum University, Chengdu 610500, China; 4 School of Computer Science, Northwestern Polytechnical University, Xi’an 710072, China

**Keywords:** memristor-based complex-valued neural networks, exponential stability, time delays

## Abstract

In this paper, the problem of stability analysis for memristor-based complex-valued neural networks (MCVNNs) with time-varying delays is investigated extensively. This paper focuses on the exponential stability of the MCVNNs with time-varying delays. By means of the Brouwer’s fixed-point theorem and *M*-matrix, the existence, uniqueness, and exponential stability of the equilibrium point for MCVNNs are studied, and several sufficient conditions are obtained. In particular, these results can be applied to general MCVNNs whether the activation functions could be explicitly described by dividing into two parts of the real parts and imaginary parts or not. Two numerical simulation examples are provided to illustrate the effectiveness of the theoretical results.

## 1. Introduction

In the past few decades, complex-valued neural networks (CVNNs) which extend the real-valued neural network (RVNNs) have aroused widespread concern because of their extensive application in various fields, such as engineering optimization, electromagnetic wave imaging, pattern recognition, and so forth [1,2]. Some conclusions about CVNNs have been obtained in [3,4]. Since the physical implementation of the nanoscale memristor in 2008 [5], memristor-based neural networks (MNNs) have attracted a remarkable amount of attention [6,7,8,9,10,11], owing to their memory characteristics and nanometer dimensions. Therefore, it is important to research the properties of MNNs which play a significant role in the system design. There exist many research results concerning the existence, uniqueness, and stability for the equilibrium of MNNs [12,13,14,15].

Figure Compared with real-valued neural networks, the complex-valued neural network (CVNN) is a frame that processes information in the complex plane—namely, their input and output signals, state variables, connection weights, and activation functions are all complex-valued [16]. In recent years, the MCVNNs which replace the real-valued MNNs (RVMNNs) in the VLSI circuits have attracted numerous researchers to study the properties of MCVNNs [17,18]. Nevertheless, it is complicated to investigate the stability of MCVNNs, since the states and the connected weights are complex-valued. In [17,18], the *n*-dimensional MCVNNs were converted into 2n-dimensional RVMNNs, and some sufficient conditions have been derived aiming to guarantee the existence, uniqueness, and exponential stability of the equilibrium. Nevertheless, not every activation functions could be explicitly described by dividing into two parts, i.e., the real part and the imaginary one. There are a few results to be applied to general MCVNNs where activation functions cannot explicitly separate the real parts and imaginary parts.

Figure Undoubtedly, due to the limited switching speed of the amplifier and the transmission delay during communication between neurons, time delays are inevitably encountered in the neural network, and the presence of time delays may cause instability or oscillation to the neural network. Therefore, it is meaningful to discuss the dynamics of neural networks with time delays [11,19,20].

Motivated by the above analysis, the exponential stability problem of MCVNNs with time-varying delays is investigated in this paper. Novel MCVNNs with time-varying delays is first presented. with the adoption of Brouwer’s fixed-point theorem, some sufficient conditions of the existence and uniqueness of the equilibrium point are achieved. Then, based on the properties of the *M*-matrix, a sufficient condition is obtained to guarantee the exponential stability for the MCVNNs with time delays. Among these sufficient conditions, the condition of the activation functions is relaxed, not to be divided into real parts and imaginary parts, but only to meet the Lipschitz condition. Therefore, the obtained method in this paper is more general than that in [17,18].

The rest of the paper is outlined as follows: in Section 2, the preliminaries, including some lemmas and necessary definitions, are stated, and the model of the MCVNNs is described; in Section 3, some sufficient conditions are achieved about the existence and the uniqueness of the equilibrium point, and several criteria are obtained to guarantee the exponential stability for the MCVNNs with time delays, while two examples are presented in Section 4.

**Notation:** The solutions of all the systems are considered in the sense of Filippov [21]. Let C and R be the sets of complex numbers and real numbers, respectively. $C^{n}$, $R^{n}$ and $R_{+}^{n}$ denote the *n*-dimensional complex, and the real and positive real vector space. z=a+ib indicates a complex number, and $\bar{z}=a+i\left(-b\right)$ denotes the conjugate number of *z*, where a,b∈R, $i=\sqrt{-1}$, $\left|z\right|=\sqrt{a^{2}+b^{2}}$. If $z={\left(z_{1},\ldots ,z_{n}\right)}^{T}\in C^{n}$, then $\left[|z|\right]=\left(\right|z_{1}\left|,\right|z_{2}\left|,\ldots ,\right|z_{n}{\left|\right)}^{T}\in R^{n}$.

## 2. Preliminaries

In this section, we will construct a class of memristor-based complex-valued neural networks, which is described as follows:

(1) $$\begin{array}{c} \frac{dz_{p}\left(t\right)}{dt}=-d_{p}z_{p}\left(t\right)+\sum_{q=1}^{n}a_{pq}\left(z_{p}\left(t\right)\right)f_{q}\left(z_{q}\left(t\right)\right)+\sum_{q=1}^{n}b_{pq}\left(z_{q}\left(t-\tau_{q}\left(t\right)\right)\right)g_{q}\left(z_{q}\left(t-\tau_{q}\left(t\right)\right)\right)+J_{p}, \end{array}$$

where p=1,2,…,n, $z_{p}\left(t\right)=x_{p}\left(t\right)+iy_{p}\left(t\right)\in C$, $d_{p}>0$ denotes the neuron self-inhibitions, $\tau_{q}\left(t\right)$ (q=1,…,n) are the transmission delays that satisfy $0\leq \tau_{q}\left(t\right)\leq \tau_{\max}$, where $\tau_{\max}$ indicates the upper bound of the delays.

Then, (1) could be rewritten equivalently in the matrix form being illustrated as follows:

(2) $$\begin{array}{c} \frac{dZ(t)}{dt}=-\Lambda Z\left(t\right)+A\left(Z\left(t\right)\right)F\left(Z\left(t\right)\right)+B\left(Z\left(t\right)\right)G\left(Z\left(t-\tau \left(t\right)\right)\right)+J, \end{array}$$

where $Z\left(t\right)={\left(z_{1}\left(t\right),\ldots ,z_{n}\left(t\right)\right)}^{T}\in C^{n}$ represents the state vector; $\Lambda =diag\{d_{1},\ldots ,d_{n}\}$; $f_{q}\left(\cdot \right)$ and $g_{q}\left(\cdot \right)$ indicate the complex-valued activation functions respectively; $F\left(Z\left(t\right)\right)={\left(f_{1}\left(z_{1}\left(t\right)\right),f_{2}\left(z_{2}\left(t\right)\right),\ldots ,f_{n}\left(z_{n}\left(t\right)\right)\right)}^{T}$ and $G\left(Z\left(t-\tau \left(t\right)\right)\right)={\left(g_{1}\left(z_{1}\left(t-\tau_{1}\left(t\right)\right)\right),\ldots ,g_{n}\left(z_{n}\left(t-\tau_{n}\left(t\right)\right)\right)\right)}^{T}$; $A\left(Z\left(t\right)\right)={\left[a_{pq}\left(z_{q}\left(t\right)\right)\right]}_{n\times n}$ and $B\left(Z\left(t\right)\right)={\left[b_{pq}\left(z_{q}\left(t\right)\right)\right]}_{n\times n}$; $J={\left[J_{1},\ldots ,J_{n}\right]}^{T}\in C^{n}$ denotes an external input vector.

**Remark** **1.** *When both the activation functions, $f_{q}$ and $g_{q}$, are real functions which can be defined by $f_{q}\left(s\right)=g_{q}\left(s\right)=\left(|s+1|-|s-1|\right)/2$, MCVNN (1) becomes the one studied in [22]; if $\tau_{pq}=0$, MCVNN (1) is degenerated, the model is investigated in [18]; when the connection weight matrices A and B are independent of the feedback states, MCVNN (1) is reduced to CVNNs with delays investigated in [23,24]. Therefore, the model in this paper is more general than than those proposed in previous literature, and all the results in the following are applicable to those special cases.*

According to the properties of the memristor, the complex-valued connection weights $a_{pq}\left(z_{q}\left(t\right)\right)$ and $b_{pq}\left(z_{q}\left(t\right)\right)$ could be described as follows:

(3) $$a_{pq}\left(z_{q}\left(t\right)\right)={\mathrm{sign}}_{pq}\frac{M_{pq}}{C_{p}}=\left\{\begin{array}{cc} {\hat{a}}_{pq}, & |z_{q}\left(t\right)|\leq 1\\ {\overset{ˇ}{a}}_{pq}, & |z_{q}\left(t\right)|>1 \end{array}\right.$$

(4) $$b_{pq}\left(z_{q}\left(t\right)\right)={\mathrm{sign}}_{pq}\frac{N_{pq}}{C_{p}}=\left\{\begin{array}{cc} {\hat{b}}_{pq}, & |z_{q}\left(t\right)|\leq 1\\ {\overset{ˇ}{b}}_{pq}, & |z_{q}\left(t\right)|>1 \end{array}\right.$$

where $M_{pq}$ and $N_{pq}$ represent the memductances of memristors $G_{pq}$ and $H_{pq}$, respectively, $G_{pq}$ stands for the memristor between the activation function $f_{q}\left(z_{q}\left(t\right)\right)$ and $z_{q}\left(t\right)$, $H_{pq}$ denotes the memristor between $g_{q}\left(z_{q}\left(t-\tau_{q}\left(t\right)\right)\right)$ and $z_{q}\left(t\right)$, $C_{p}$ represents the capacitor, and ${\mathrm{sign}}_{pq}$ represents the sign function, which is provided as

(5) $${\mathrm{sign}}_{pq}=\left\{\begin{array}{cc} 1, & p\neq q,\\ -1, & p=q, \end{array}\right.$$

where the complex-valued constants ${\hat{a}}_{pq},{\overset{ˇ}{a}}_{pq},{\hat{b}}_{pq},{\overset{ˇ}{b}}_{pq}$ are the switching jumps.

Next, we will introduce some useful definitions and assumptions.

**Definition** **1.** *Let $E\subset C^{n}$, x↦F(x) be a set-valued map from $E↪C^{n}$, if there exists a nonempty set $F\left(x\right)\subset C^{n}$ for any point $x\in E\subset C^{n}$. A nonempty set-valued map F is upper-semi-continuous at $x_{0}\in E\subseteq C^{n}$, if, for any open set N containing $F(x_{0})$, there exists a neighborhood M of $x_{0}$ such that F(M)⊂N. F(x) is called a closed (convex, compact) image if for all x∈E.*

**Definition** **2.** *For $\frac{dx}{dt}=f\left(t,x\right)$, $x\in C^{n}$, where f(t,x) is discontinuous in x and the set-valued map of f(t,x) is defined as:*

(6) $$F\left(t,x\right)=\underset{\delta >0}{⋂}\underset{\mu (N)=0}{⋂}\mathrm{co}\left[f\left(B\left(x,\delta \right)\backslash N\right)\right],$$

*where B(x,δ)={y:|y−x|≤δ} is the ball with a center x and radius δ; and the intersection is applied to all sets N of measure zero and all δ>0; while μ(N) denotes the Lebesgue measure of set N. A Filippov solution of the Cauchy problem with initial condition $x\left(0\right)=x_{0}$ is absolutely continuous on any subinterval $t\in [t_{1},t_{2}]$ of [0,T], which satisfies $x\left(0\right)=x_{0}$ and the differential inclusion:*

(7) $$\frac{dx}{dt}\in F\left(t,x\right),\;\mathrm{for}\;a.a.\;t\in \left[0,T\right].$$

In this paper, $a_{pq}\left(z_{p}\left(t\right)\right)$ and $b_{pq}\left(z_{q}\left(t-\tau_{q}\left(t\right)\right)\right)$ are dependent on the states, and they are discontinuous. Therefore, the solutions of all systems are intended in Filippov’s sense.

Under Definition 1, (1) could be rewritten as follows:

(8) $$\begin{array}{c} \frac{dz_{p}\left(t\right)}{dt}\in -d_{p}z_{p}\left(t\right)+\sum_{q=1}^{n}co\left\{{\hat{a}}_{pq},{\overset{ˇ}{a}}_{pq}\right\}f_{q}\left(z_{q}\left(t\right)\right)+\sum_{q=1}^{n}co\left\{{\hat{b}}_{pq},{\overset{ˇ}{b}}_{pq}\right\}g_{q}\left(z_{q}\left(t-\tau_{q}\left(t\right)\right)\right)+J_{p}, \end{array}$$

or equivalently, for all p,q∈{1,2,…,n},t≥0, there exit measurable functions ${\tilde{a}}_{pq}\left(t\right)\in co\left\{{\hat{a}}_{pq},{\overset{ˇ}{a}}_{pq}\right\}$ and ${\tilde{b}}_{pq}\left(t\right)\in co\left\{{\hat{b}}_{pq},{\overset{ˇ}{b}}_{pq}\right\}$ such that

(9) $$\begin{array}{c} \frac{dz_{p}\left(t\right)}{dt}=-d_{p}z_{p}\left(t\right)+\sum_{q=1}^{n}{\tilde{a}}_{pq}\left(t\right)f_{q}\left(z_{q}\left(t\right)\right)+\sum_{q=1}^{n}{\tilde{b}}_{pq}\left(t\right)g_{q}\left(z_{q}\left(t-\tau_{q}\left(t\right)\right)\right)+J_{p}, \end{array}$$

Then, (9) could be transformed into the matrix format, which is provided as follows

(10) $$\begin{array}{c} \frac{dZ(t)}{dt}=-\Lambda Z\left(t\right)+\tilde{A}\left(t\right)F\left(Z\left(t\right)\right)+\tilde{B}\left(t\right)G\left(Z\left(t-\tau \left(t\right)\right)\right)+J, \end{array}$$

where $\tilde{A}\left(t\right)={\left[{\tilde{a}}_{pq}\left(t\right)\right]}_{n\times n}\in C^{n\times n}$ and $\tilde{B}\left(t\right)={\left[{\tilde{b}}_{pq}\left(t\right)\right]}_{n\times n}\in C^{n\times n}$.

Before giving our main results, an assumption should be given.

**Assumption** **1.** *For q=1,…,n, the Lipschitz continuity condition of the activation functions $f_{q}\left(\cdot \right)$ and $g_{q}\left(\cdot \right)$ should be satisfied in the complex field—that is, there exist constants $l_{q}^{f}>0$ and $l_{q}^{g}>0$, such that, for any $z^{1},z^{2}\in C$, we have*

(11) $$|f_{q}\left(z^{1}\right)-f_{q}\left(z^{2}\right)|\leq l_{q}^{f}|z^{1}-z^{2}\left|,\right|g_{q}\left(z^{1}\right)-g_{q}\left(z^{2}\right)|\leq l_{q}^{g}\left|z^{1}-z^{2}\right|$$

*where $l_{q}^{f}$ and $l_{q}^{g}$ denote Lipschitz constants, respectively.*

**Remark** **2.** *In [18,25,26], it is necessary to ensure that the activation functions can be explicitly expressed by separating into real and imaginary parts, which is provided as*

(12) $$f_{q}\left(z\left(t\right)\right)=f_{q}^{R}\left(x\left(t\right),y\left(t\right)\right)+if_{q}^{I}\left(x\left(t\right),y\left(t\right)\right)$$

*where $f_{q}^{R}\left(\cdot ,\cdot \right):R^{2}\to R$ and $f_{q}^{I}\left(\cdot ,\cdot \right):R^{2}\to R$ denote the real and imaginary parts of $f_{q}\left(\cdot \right)$, respectively. In addition, it is always required that $\frac{\partial f_{j}^{R}}{\partial x_{j}},\frac{\partial f_{j}^{R}}{\partial y_{j}},\frac{\partial f_{j}^{I}}{\partial x_{j}},and\frac{\partial f_{j}^{I}}{\partial y_{j}}$ are existent, continuous, and bounded, aiming to guarantee the stability of the system considered in [27]. In fact, these necessary conditions are conservative, since not every activation function could be explicitly separated into real parts and imaginary parts. In this paper, $f_{q}\left(\cdot \right)$ and $g_{q}\left(\cdot \right)$ are only necessary in order to satisfy Assumption 1. Moreover, if the conditions in [18,25,26], the activation functions $f_{q}\left(\cdot \right)$ and $g_{q}\left(\cdot \right)$ could satisfy the Assumption 1. Hence, the obtained results seem to be more general and less conservative than those which appeared in [17,18,25,26,27].*

**Definition** **3.** *For any given initial time $t_{0}\in R$, the complex-valued function $Z\left(t\right)\in C[\left[t_{0}-\tau ,+\infty \right),C^{n}]$ is designated a solution of (10) through $\left(t_{0},\phi \right)$, if Z(t) satisfies the initial condition*

(13) $$Z\left(t_{0}+s\right)=\phi \left(s\right),\;\;s\in \left[-\tau ,0\right],$$

for $t\geq t_{0}$, denoted by $Z(t,t_{0},\phi )$ (or Z for short). Particularly, a point $Z{\left(t\right)}^{*}\in C^{n}$ is named an equilibrium point of (10), if $Z\left(t\right)=Z^{*}$ is the solution of (10).

**Lemma** **1.** *[24]: Let $P={\left(p_{ij}\right)}_{n\times n}$ with $p_{ij}\geq 0$ for i≠j and $Q={\left(q_{ij}\right)}_{n\times n}\geq 0$. Suppose −(P+Q) be an M-matrix. For any time $b\in (t_{0},+\infty )$, let $u\left(t\right)={\left(u_{1}\left(t\right),\ldots ,u_{n}\left(t\right)\right)}^{T}\in C\left(\left[t_{0},b\right),R_{+}^{n}\right)$ satisfies the following delay differential inequality for any initial condition $u\left(s\right)\in C(\left[t_{0}-\tau ,t_{0}\right],R_{+}^{n})$:*

(14) $$D^{+}u\left(t\right)\leq Pu\left(t\right)+Q{\left[|u\left(t\right)|\right]}_{\tau }$$

*where $t\geq t_{0}$, ${\left[|u\left(t\right)|\right]}_{\tau }=\left(\right|u_{1}{\left(t\right)|}_{\tau },\ldots ,|u_{n}\left(t\right){{|}_{\tau })}^{T}$, $|u_{p}{\left(t\right)|}_{\tau }=\sup_{-\tau \leq s\leq 0}\left|u_{p}\left(t+s\right)\right|$ for p=1,2,…,n. Then, $u\left(t\right)\leq \xi e^{-\lambda (t-t_{0})},\;t\geq t_{0}$, as long as $u\left(s\right)\leq \xi e^{-\lambda (s-t_{0})},\;t_{0}-\tau \leq s\leq t_{0}$, where $\xi ={\left(\xi_{1},\ldots ,\xi_{n}\right)}^{T}$ is a positive real vector, and λ>0 is decided by the inequality: $(\lambda I+P+Qe^{\lambda \tau })\xi <0$.*

**Definition** **4.** [24] The equilibrium point $z^{*}$ of (8) is said to be exponentially stable when there are constants λ>0 and M≥1, such that for all $t\geq t_{0}$ the inequality $|Z\left(t\right)-Z^{*}|\leq M|\phi \left(s\right)-Z^{*}|e^{-\lambda (t-t_{0})}$ is satisfied.

## 3. Main Results

In the following, we will firstly propose several sufficient conditions to ensure that (1) has a unique equilibrium point, and then corresponding proof is provided, aiming to ensure that the unique equilibrium point is global exponentially stable.

**Theorem** **1.** Suppose Assumption 1 is satisfied and $\Lambda -\Phi L_{f}-\Psi L_{g}$ is an M-matrix, there is a unique equilibrium point $Z^{*}$ for (1), where $\Phi ={\left(w_{pq}\right)}_{n\times n}$ with $w_{pq}=\sup_{t\geq 0}\left(\right|{\tilde{a}}_{pq}\left(t\right)\left|\right)$ and $\Psi ={\left(v_{pq}\right)}_{n\times n}$ with $v_{pq}=\sup_{t\geq 0}\left(\right|{\tilde{b}}_{pq}\left(t\right)\left|\right)$, $L_{f}=\mathrm{diag}\left\{l_{1}^{f},\ldots ,l_{n}^{f}\right\}$ and $L_{g}=\mathrm{diag}\left\{l_{1}^{g},\ldots ,l_{n}^{g}\right\}$.

**Proof.**  Firstly, we will try to illustrate that (10) has an equilibrium point $Z^{*}$—that is, we should prove $Z^{*}$ is a solution of the following equation

(15) $$\begin{array}{c} -\Lambda Z+\tilde{A}\left(t\right)F\left(Z\right)+\tilde{B}\left(t\right)G\left(Z\right)+J=0. \end{array}$$

Consider the following operator according to the differential Equation (9)

(16) $$\begin{array}{c} H_{p}\left(z\right)=d_{p}^{-1}\left(\sum_{q=1}^{n}{\tilde{a}}_{pq}f_{q}\left(z_{q}\right)+\sum_{q=1}^{n}{\tilde{b}}_{pq}g_{q}\left(z_{q}\right)+J_{p}\right). \end{array}$$

Then, (16) could be transformed into the following matrix format:

(17) $$\begin{array}{c} H\left(Z\right)=\Lambda^{-1}\left(\tilde{A}F\left(Z\right)+\tilde{B}G\left(Z\right)+J\right), \end{array}$$

where $z\in C^{n}$, $H\left(Z\right)={\left(H_{1}\left(z\right),\ldots ,H_{n}\left(z\right)\right)}^{T}$, $F\left(Z\right)={\left(f_{1}\left(z_{1}\right),\ldots ,f_{n}\left(z_{n}\right)\right)}^{T}$ and $G\left(Z\right)={\left(g_{1}\left(z_{1}\right),\ldots ,g_{n}\left(z_{n}\right)\right)}^{T}$.

with the adoption of Assumption 1, one has

(18) $$\begin{array}{ccc} |H_{p}\left(z\right)| & \leq & d_{p}^{-1}(\sum_{q=1}^{n}|{\tilde{a}}_{pq}\left|\right|f_{q}\left(z_{q}\right)|+\sum_{q=1}^{n}|{\tilde{b}}_{pq}\left|\right|g_{q}\left(z_{q}\right)\left|+\right|J_{p}\left|\right)\\ & = & d_{p}^{-1}(\sum_{q=1}^{n}|{\tilde{a}}_{pq}\left|\right|f_{q}\left(z_{q}\right)-f_{q}\left(0\right)+f_{q}\left(0\right)|+\sum_{q=1}^{n}|{\tilde{b}}_{pq}\left|\right|g_{q}\left(z_{q}\right)-g_{q}\left(0\right)+g_{q}\left(0\right)\left|+\right|J_{p}\left|\right)\\ & \leq & d_{p}^{-1}(\sum_{q=1}^{n}|{\tilde{a}}_{pq}\left|(\right|f_{q}\left(z_{q}\right)-f_{q}\left(0\right)\left|+\right|f_{q}\left(0\right)\left|\right)+\sum_{q=1}^{n}|{\tilde{b}}_{pq}\left|(\right|g_{q}\left(z_{q}\right)-g_{q}\left(0\right)\left|+\right|g_{q}\left(0\right)\left|)+\right|J_{p}\left|\right)\\ & \leq & d_{p}^{-1}(\sum_{q=1}^{n}w_{pq}\left(\right|f_{q}\left(z_{q}\right)-f_{q}\left(0\right)\left|\right)+\sum_{q=1}^{n}v_{pq}\left(\right|g_{q}\left(z_{q}\right)-g_{q}\left(0\right)\left|)+\right|{\tilde{J}}_{p}\left|\right)\\ & \leq & d_{p}^{-1}(\sum_{q=1}^{n}w_{pq}l_{q}^{f}|z_{q}|+\sum_{q=1}^{n}v_{pq}l_{q}^{g}|z_{q}\left|+\right|{\tilde{J}}_{p}\left|\right) \end{array}$$

where $|{\tilde{J}}_{p}|=\sum_{q=1}^{n}w_{pq}|f_{q}\left(0\right)|+\sum_{q=1}^{n}v_{pq}|g_{q}\left(0\right)|+|J_{p}|$. Then, one can get

(19) $$\begin{array}{c} \left[|H\left(Z\right)|\right]\leq \Lambda^{-1}(\left(\Phi L_{f}+\Psi L_{g}\right)\left[|Z|\right]+\left[\right|\tilde{J}|]), \end{array}$$

where $\left[|H\left(z\right)|\right]=\left(\right|H_{1}\left(z\right)|,\ldots ,|H_{1}\left(z\right){\left|\right)}^{T}$, $\left[|Z|\right]=\left(\right|z_{1}\left|,\ldots ,\right|z_{n}{\left|\right)}^{T}$ and $\left[\right|\tilde{J}\left|\right]=\left(\right|{\tilde{J}}_{1}\left|,\ldots ,\right|{\tilde{J}}_{n}{\left|\right)}^{T}$. Since $\Lambda -\Phi L_{f}-\Psi L_{g}$ is an *M*-matrix, there is a positive vector $\xi \in R^{n}$ such that

$$\left[\right|\tilde{J}\left|\right]\leq \left(\Lambda -\Phi L_{f}-\Psi L_{g}\right)\xi ,$$

yielding

$$\Lambda^{-1}\left[\right|\tilde{J}\left|\right]\leq \left(I-\Lambda^{-1}\left(\Phi L_{f}+\Psi L_{g}\right)\right)\xi ,$$

or

$$\Lambda^{-1}\left(\left(\Phi L_{f}+\Psi L_{g}\right)\right)\xi +\left[\right|\tilde{J}\left|]\right)\leq \xi .$$

Consider $B=\{Z\in C^{n}\left|[|Z|]\leq \xi \right\}$. Therefore, for any Z∈B, one has [|H(Z)|]≤ξ. That is, the continuous operator H maps convex and compact set B into B. According to Brouwer’s fixed-point theorem, *H* has a fixed point $z^{*}\in B$, and $z^{*}$ is also the equilibrium point of (10).

Next, we will prove the equilibrium point $Z^{*}\in B$ of (10) is unique. By means of apagoge, if it is not true, there exists another equilibrium point $Z^{**}\in B$ with $Z^{*}\neq Z^{**}$. Then,

$$Z^{*}=\Lambda^{-1}\left(\tilde{A}F\left(Z^{*}\right)+\tilde{B}G\left(Z^{*}\right)+J\right),\;\;z^{**}=\Lambda^{-1}\left(\tilde{A}F\left(Z^{**}\right)+\tilde{B}G\left(Z^{**}\right)+J\right)$$

As a result, one has $|Z^{*}-Z^{**}|\leq \Lambda^{-1}\left(\Phi L_{f}+\Psi L_{g}\right)\left|Z^{*}-Z^{**}\right|$. When $Z^{*}\neq Z^{**}$, we have $[|Z^{*}-Z^{**}|]>0$. Then, one has $\rho (\Lambda^{-1}\left(\Phi L_{f}+\Psi L_{g}\right))\geq 1$. On the other hand, $\Lambda -\Phi L_{f}-\Psi L_{g}$ is an *M*-matrix, which implies that $\rho (\Lambda^{-1}\left(\Phi L_{f}+\Psi L_{g}\right))\leq 1$. This contradiction indicates that $Z^{*}=Z^{**}$—that is, there exists a unique equilibrium point for (10). □

**Theorem** **2.** Suppose Assumption 1 holds. If the condition in Theorem 1 is satisfied, the unique equilibrium point $Z^{*}$ is global exponentially stable.

**Proof.**  Under Assumption 1, there exists a unique equilibrium point for (10) if the condition in Theorem 1 is satisfied. Suppose $Z^{*}$ is the unique equilibrium point of (10). Then, using the translation $\tilde{Z}\left(t\right)=Z\left(t\right)-Z^{*}$, we can transfer the equilibrium point to the origin. Hence, we obtain

(20) $$\begin{array}{c} \frac{d\tilde{Z}\left(t\right)}{dt}=-\Lambda \tilde{Z}\left(t\right)+\tilde{A}\left(t\right)\tilde{F}\left(\tilde{Z}\left(t\right)\right)+\tilde{B}\left(t\right)\tilde{G}\left(\tilde{Z}\left(t-\tau \left(t\right)\right)\right),\;t\geq 0 \end{array}$$

where $\tilde{Z}\left(t\right)={\left({\tilde{z}}_{1}\left(t\right),\ldots ,{\tilde{z}}_{n}\left(t\right)\right)}^{T}$, $\tilde{F}\left(\tilde{Z}\left(t\right)\right)=F\left(\tilde{Z}\left(t\right)+Z^{*}\right)-F\left(Z^{*}\right)$ and $\tilde{G}\left(\tilde{Z}\left(t-\tau \left(t\right)\right)\right)=G\left(\tilde{Z}\left(t-\tau \left(t\right)\right)+Z^{*}\right)-G\left(Z^{*}\right)$.

By using (19), we can have

(21) $$\begin{array}{ccc} \frac{d}{dt}\left[\right|\tilde{Z}\left(t\right){\left|\right]}^{2} & = & 2Re({\tilde{Z}}^{H}\left(t\right)\dot{\tilde{Z}}\left(t\right))\\ & = & 2Re({\tilde{Z}}^{H}\left(t\right)\left(-\Lambda \tilde{Z}\left(t\right)+\tilde{A}\left(t\right)\tilde{F}\left(\tilde{Z}\left(t\right)\right)+\tilde{B}\left(t\right)\tilde{G}\left(\tilde{Z}\left(t-\tau \left(t\right)\right)\right)\right))\\ & = & -2Re\left({\tilde{Z}}^{H}\left(t\right)\Lambda \tilde{Z}\left(t\right)\right)+2Re\left({\tilde{Z}}^{H}\left(t\right)\tilde{A}\left(t\right)\tilde{F}\left(\tilde{Z}\left(t\right)\right)\right)+2Re\left({\tilde{Z}}^{H}\left(t\right)\tilde{B}\left(t\right)\tilde{G}\left(\tilde{Z}\left(t-\tau \left(t\right)\right)\right)\right)) \end{array}$$

where $\left[\right|\tilde{Z}\left(t\right)|]=\left(\right|{\tilde{z}}_{1}\left(t\right)|,\ldots ,|{\tilde{z}}_{n}\left(t\right){\left|\right)}^{T}$ and $\left[\right|\tilde{Z}{\left(t\right)|]}^{2}=\left(\right|{\tilde{z}}_{1}{\left(t\right)|}^{2},\ldots ,|{\tilde{z}}_{n}\left(t\right){{|}^{2})}^{T}$.

Because Λ is a real diagonal matrix $\Lambda =diag\{d_{1},\ldots ,d_{n}\}$, one has

(22) $$Re\left({\tilde{Z}}^{H}\left(t\right)\Lambda \tilde{Z}\left(t\right)\right)=\sum_{p=1}^{n}d_{p}{\bar{\tilde{z}}}_{p}\left(t\right){\tilde{z}}_{p}\left(t\right)=\left[\right|\tilde{Z}{\left(t\right)|]}^{T}\Lambda [|\tilde{Z}\left(t\right)\left|\right],$$

where ${\bar{\tilde{z}}}_{p}\left(t\right)$ is the conjugator of ${\tilde{z}}_{p}\left(t\right)$.

According to Assumption 1, we could get

(23) $$\begin{array}{ccc} Re({\tilde{Z}}^{H}\left(t\right)\tilde{A}\left(t\right)\tilde{F}\left(\tilde{z}\left(t\right)\right)) & = & Re\left(\sum_{p=1}^{n}\sum_{q=1}^{n}{\tilde{a}}_{pq}\left(t\right){\bar{\tilde{z}}}_{p}\left(t\right){\tilde{f}}_{q}\left({\tilde{z}}_{q}\left(t\right)\right)\right)\leq \sum_{p=1}^{n}\sum_{q=1}^{n}|{\tilde{a}}_{pq}\left(t\right)||{\bar{\tilde{z}}}_{p}\left(t\right)||{\tilde{f}}_{q}\left({\tilde{z}}_{q}\left(t\right)\right)|\\ & \leq & \sum_{p=1}^{n}\sum_{q=1}^{n}|w_{pq}\left(t\right)|l_{q}^{f}|{\tilde{z}}_{p}\left(t\right)||{\tilde{z}}_{q}\left(t\right)|=[|\tilde{Z}{\left(t\right)|]}^{T}\Phi L_{f}\left[\right|\tilde{Z}\left(t\right)\left|\right] \end{array}$$

Similar with (23), we have

(24) $$\begin{array}{ccc} Re({\tilde{Z}}^{H}\left(t\right)\tilde{B}\left(t\right)\tilde{G}\left(\tilde{Z}\left(t-\tau \left(t\right)\right)\right)) & = & Re(\sum_{p=1}^{n}\sum_{q=1}^{n}{\tilde{b}}_{pq}\left(t\right){\bar{\tilde{z}}}_{p}\left(t\right){\tilde{g}}_{q}\left({\tilde{z}}_{q}\left(t-\tau \left(t\right)\right)\right))\\ & \leq & \sum_{p=1}^{n}\sum_{q=1}^{n}|{\tilde{b}}_{pq}\left(t\right)||{\bar{\tilde{z}}}_{p}\left(t\right)||{\tilde{g}}_{q}\left({\tilde{z}}_{q}\left(t-\tau \left(t\right)\right)\right)|\\ & \leq & \sum_{p=1}^{n}\sum_{q=1}^{n}|v_{pq}\left(t\right)|l_{q}^{g}|{\tilde{z}}_{p}\left(t\right)||{\tilde{z}}_{q}\left(t-\tau \left(t\right)\right)|\\ & = & \left[\right|\tilde{Z}{\left(t\right)|]}^{T}\Psi L_{g}\left[\right|\tilde{Z}\left(t-\tau \left(t\right)\right)\left|\right] \end{array}$$

Combining (21), (22), and (23) into (20) and noticing that $\frac{d}{dt}\left[\right|\tilde{Z}{\left(t\right)|]}^{2}=2[|\tilde{Z}{\left(t\right)|]}^{T}\frac{d}{dt}\left[\right|\tilde{Z}\left(t\right)\left|\right]$, one gets

(25) $$\begin{array}{c} 2[|\tilde{Z}{\left(t\right)|]}^{T}\frac{d}{dt}\left[\right|\tilde{Z}\left(t\right)|]\leq -2[|\tilde{Z}{\left(t\right)|]}^{T}\left(\Lambda [\right|\tilde{Z}\left(t\right)|]-\Phi L_{f}\left[\right|\tilde{Z}\left(t\right)|]-\Psi L_{g}\left[\right|\tilde{Z}\left(t-\tau \left(t\right)\right)|]),\;t\geq 0 \end{array}$$

that is,

(26) $$\begin{array}{c} \frac{d}{dt}\left[\right|\tilde{Z}\left(t\right)|]\leq -\left(\Lambda [\right|\tilde{Z}\left(t\right)|]-\Phi L_{f}\left[\right|\tilde{Z}\left(t\right)|]-\Psi L_{g}\left[\right|\tilde{Z}\left(t-\tau \left(t\right)\right)|]),\;t\geq 0 \end{array}$$

Since $\Lambda -\Phi L_{f}-\Psi L_{g}$ is an *M*-matrix, there exists a vector ξ such that $(\Lambda -\Phi L_{f}-\Psi L_{g})\xi >0$.

For any given initial condition $\tilde{\phi }\left(s\right)=\phi \left(s\right)-Z^{*}$, s∈[−τ,0], by Lemma 1, we could obtain

(27) $$\begin{array}{c} \left[\right|\tilde{Z}\left(t\right)|]\leq \Theta [|\tilde{\phi }\left(s\right){|}_{\tau }]e^{-\lambda (t-t_{0})},\;t\geq t_{0} \end{array}$$

where $\Theta =\max\{1,\frac{\max_{1\leq k\leq n}\left\{\xi_{k}\right\}}{\min_{1\leq k\leq n}\left\{\right|{\tilde{\phi }}_{k}{|}_{\tau }\}}\}$, λ is decided by the inequality $(\lambda I-\Lambda +\Phi L_{f}+\Psi L_{g}e^{\lambda \tau })\xi <0$. This leads to the result

(28) $$\begin{array}{c} |\tilde{Z}\left(t\right)|\leq \Theta [|\tilde{\phi }\left(s\right){|}_{\tau }]e^{-\lambda (t-t_{0})},\;t\geq t_{0} \end{array}$$

The proof completes. □

Figure In this section, some sufficient conditions are achieved about the existence and uniqueness of the equilibrium point, and several criteria are obtained to guarantee the exponential stability for the MCVNNs with time delays. These results obtained can be applied to more general MCVNNs whether the activation functions are explicitly described by either dividing the real parts and imaginary parts, or not.

## 4. Examples

In this section, two examples are given to demonstrate the validity of the obtained results.

**Example** **1.** *Consider a two-order MCVNN, as follows:*

(29) $$\begin{array}{c} \frac{dz_{p}\left(t\right)}{dt}=-d_{p}z_{p}\left(t\right)+\sum_{q=1}^{2}a_{pq}\left(z_{p}\left(t\right)\right)f_{q}\left(z_{q}\left(t\right)\right)+\sum_{q=1}^{2}b_{pq}\left(z_{q}\left(t-\tau_{q}\left(t\right)\right)\right)g_{q}\left(z_{q}\left(t-\tau_{q}\left(t\right)\right)\right)+J_{p},p=1,2, \end{array}$$

*where $z_{p}\left(t\right)=x_{p}\left(t\right)+iy_{p}\left(t\right)$, $d_{1}=5,d_{2}=6$, $J_{1}=1.5-2.5i,J_{2}=-1-0.5i$ and the time delays $\tau_{q}\left(t\right)=\frac{e^{t}}{1+e^{t}}$,*

*$a_{11}\left(z_{1}\left(t\right)\right)=\left\{\begin{array}{cc} -1.8+2i, & |z_{1}\left(t\right)|\leq 1,\\ -1.5+2i, & |z_{1}\left(t\right)|>1, \end{array}\right.$$a_{12}\left(z_{1}\left(t\right)\right)=\left\{\begin{array}{cc} 2.8+1.2i, & |z_{1}\left(t\right)|\leq 1,\\ 2.5+i, & |z_{1}\left(t\right)|>1, \end{array}\right.$*

*$a_{21}\left(z_{2}\left(t\right)\right)=\left\{\begin{array}{cc} 1+i, & |z_{1}\left(t\right)|\leq 1,\\ 0.8+i, & |z_{1}\left(t\right)|>1, \end{array}\right.$$a_{22}\left(z_{2}\left(t\right)\right)=\left\{\begin{array}{cc} -1+i, & |z_{1}\left(t\right)|\leq 1,\\ -0.8+i, & |z_{1}\left(t\right)|>1, \end{array}\right.$*

$$b_{11}\left(z_{1}\left(t-\tau_{1}\left(t\right)\right)\right)=\left\{\begin{array}{cc} -3.5+i, & |z_{1}\left(t-\tau_{1}\left(t\right)\right)|\leq 1,\\ -3.2+i, & |z_{1}\left(t-\tau_{1}\left(t\right)\right)|>1, \end{array}\right.$$

$$b_{12}\left(z_{1}\left(t-\tau_{1}\left(t\right)\right)\right)=\left\{\begin{array}{cc} -0.5+i, & |z_{1}\left(t-\tau_{1}\left(t\right)\right)|\leq 1,\\ -0.1+i, & |z_{1}\left(t-\tau_{1}\left(t\right)\right)|>1, \end{array}\right.$$

$$b_{21}\left(x_{2}\left(t-\tau_{2}\left(t\right)\right)\right)=\left\{\begin{array}{cc} 0.1+i, & |z_{2}\left(t-\tau_{2}\left(t\right)\right)|\leq 1,\\ 0.2+i, & |z_{2}\left(t-\tau_{2}\left(t\right)\right)|>1, \end{array}\right.$$

$$b_{22}\left(z_{2}\left(t-\tau_{2}\left(t\right)\right)\right)=\left\{\begin{array}{cc} -3.6+i, & |z_{2}\left(t-\tau_{2}\left(t\right)\right)|\leq 1,\\ -3.2+i, & |z_{2}\left(t-\tau_{2}\left(t\right)\right)|>1. \end{array}\right.$$

*Therefore, one can get*

(30) $$\begin{array}{cc} \Phi =\left(\begin{array}{cc} \sqrt{7.24} & \sqrt{9.28}\\ \sqrt{2} & \sqrt{2} \end{array}\right) & \Psi =\left(\begin{array}{cc} \sqrt{13.25} & \sqrt{1.25}\\ \sqrt{1.04} & \sqrt{13.96} \end{array}\right) \end{array}$$

*Assume the activation functions of (29) as follows:*

$$f_{q}\left(z_{q}\right)=0.5|y_{q}\left|+0.5i\right|x_{q}|,\;\;g_{q}\left(z_{q}\right)=\frac{1-e^{-y_{q}}}{1+e^{-y_{q}}}+i\frac{1}{1+e^{-x_{q}}},\;\;q=1,2.$$

*Then, through simple calculation, one can get the activation functions which satisfy Assumption 1. That is, for any $z_{q}=x_{q}+iy_{q},{\tilde{z}}_{q}={\tilde{x}}_{q}+i{\tilde{y}}_{q}\in C$, one can have*

(31) $$\begin{array}{ccc} |f_{q}\left(z_{q}\right)-f_{q}\left({\tilde{z}}_{q}\right)| & = & 0.5|(|y_{q}\left|-\right|{\tilde{y}}_{q}\left|\right)+i(|x_{q}\left|-\right|{\tilde{x}}_{q}\left|)\right|\\ & = & 0.5\sqrt{\left(\right|x_{q}\left|-\right|{\tilde{x}}_{q}{\left|\right)}^{2}+\left(\right|y_{q}\left|-\right|{\tilde{y}}_{q}{\left|\right)}^{2}}\\ & \leq & 0.5\sqrt{{\left(x_{q}-{\tilde{x}}_{q}\right)}^{2}+{\left(y_{q}-{\tilde{y}}_{q}\right)}^{2}}=0.5\left|z_{q}-{\tilde{z}}_{q}\right| \end{array}$$

(32) $$\begin{array}{ccc} |g_{q}\left(z_{q}\right)-g_{q}\left({\tilde{z}}_{q}\right)| & = & |\left(\frac{1-e^{-y_{q}}}{1+e^{-y_{q}}}-\frac{1-e^{-{\tilde{y}}_{q}}}{1+e^{-{\tilde{y}}_{q}}}\right)+i\left(\frac{1}{1+e^{-x_{q}}}-\frac{1}{1+e^{-{\tilde{x}}_{q}}}\right)|\\ & = & \sqrt{{\left(\frac{1-e^{-y_{q}}}{1+e^{-y_{q}}}-\frac{1-e^{-{\tilde{y}}_{q}}}{1+e^{-{\tilde{y}}_{q}}}\right)}^{2}+{\left(\frac{1}{1+e^{-x_{q}}}-\frac{1}{1+e^{-{\tilde{x}}_{q}}}\right)}^{2}}\\ & \leq & \sqrt{0.0625{\left(x_{q}-{\tilde{x}}_{q}\right)}^{2}+0.25{\left(y_{q}-{\tilde{y}}_{q}\right)}^{2}}\leq 0.5\left|z_{q}-{\tilde{z}}_{q}\right| \end{array}$$

*Then, one has $L_{f}=L_{g}=\mathrm{diag}\left(0.5,0.5\right)$.*

**Figure 1:**
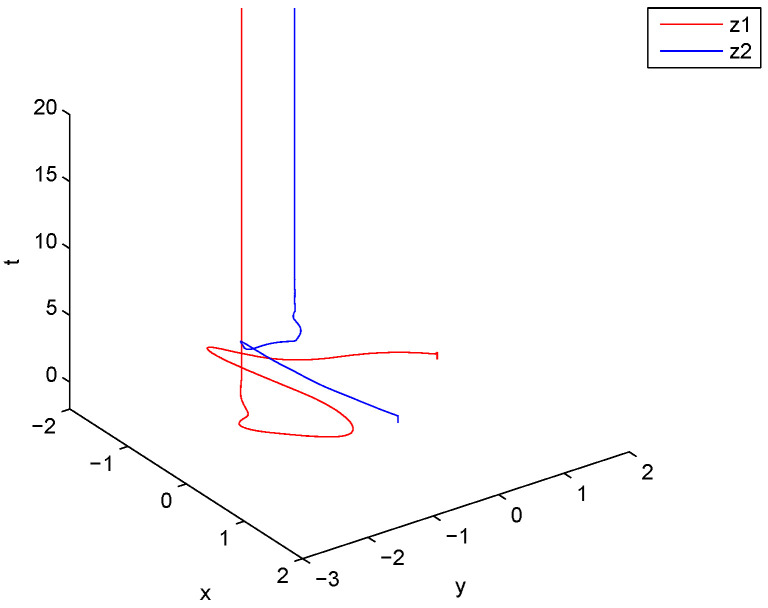
Curves of $z_{1}$ and $z_{2}$.

**Figure 2:**
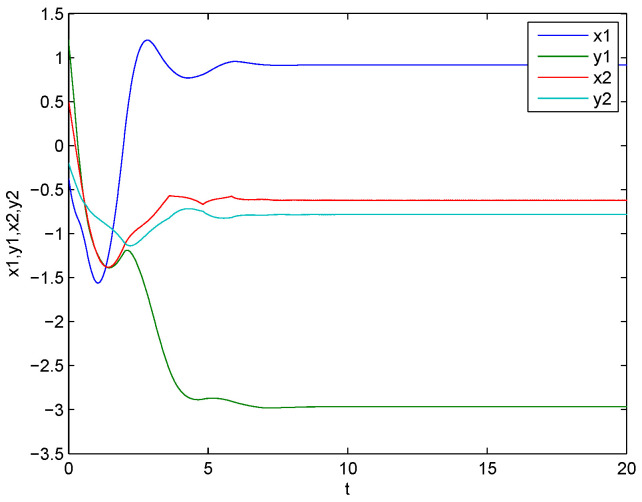
Curves of the real and imaginary parts of $z_{1}$ and $z_{2}$.

We have that

(33) $$\Lambda -\Phi L_{f}-\Psi L_{g}=\left(\begin{array}{cc} 1.8346 & -2.0822\\ -1.2170 & 3.4247 \end{array}\right)$$

is an M matrix, then the conditions of Theorem 1 are satisfied. Let the initial values of (29) be $z_{1}\left(s\right)=,z_{2}\left(s\right)=$ for s∈[−1,0]. Figure 1 and Figure 2 show that the equilibrium point of (29) is existent, unique, and exponentially stable.

**Example** **2.** *Consider the MCVNNs with $d_{1}=4,d_{2}=5$, $J_{1}=1.2-1.5i,J_{2}=-0.1-2.5i$ and the time-varying delays $\tau_{q}\left(t\right)=2+0.5sin\left(t\right)$,*

$$\begin{array}{ll} a_{11}\left(z_{1}\left(t\right)\right)=\left\{\begin{array}{cc} -1.2+0.2i, & |z_{1}\left(t\right)|\leq 1,\\ -1.0+0.2i, & |z_{1}\left(t\right)|>1, \end{array}\right. & a_{12}\left(z_{1}\left(t\right)\right)=\left\{\begin{array}{cc} 1.8+1.2i, & |z_{1}\left(t\right)|\leq 1,\\ 1.5+i, & |z_{1}\left(t\right)|>1, \end{array}\right.\\ a_{21}\left(z_{2}\left(t\right)\right)=\left\{\begin{array}{cc} 1+i, & |z_{2}\left(t\right)|\leq 1,\\ 0.8+i, & |z_{2}\left(t\right)|>1, \end{array}\right. & a_{22}\left(z_{2}\left(t\right)\right)=\left\{\begin{array}{cc} -1.2+i, & |z_{2}\left(t\right)|\leq 1,\\ -1.8+i, & |z_{2}\left(t\right)|>1, \end{array}\right. \end{array}$$

$$b_{11}\left(z_{1}\left(t-\tau_{1}\left(t\right)\right)\right)=\left\{\begin{array}{cc} -1.5+1.5i, & |z_{1}\left(t-\tau_{1}\left(t\right)\right)|\leq 1,\\ -1.2+1.2i, & |z_{1}\left(t-\tau_{1}\left(t\right)\right)|>1, \end{array}\right.$$

$$b_{12}\left(z_{1}\left(t-\tau_{1}\left(t\right)\right)\right)=\left\{\begin{array}{cc} -0.5+2i, & |z_{1}\left(t-\tau_{1}\left(t\right)\right)|\leq 1,\\ -0.3+1.8i, & |z_{1}\left(t-\tau_{1}\left(t\right)\right)|>1, \end{array}\right.$$

$$b_{21}\left(z_{2}\left(t-\tau_{2}\left(t\right)\right)\right)=\left\{\begin{array}{cc} 0.1+i, & |z_{2}\left(t-\tau_{2}\left(t\right)\right)|\leq 1,\\ 0.2+i, & |z_{2}\left(t-\tau_{2}\left(t\right)\right)|>1, \end{array}\right.$$

$$b_{22}\left(z_{2}\left(t-\tau_{2}\left(t\right)\right)\right)=\left\{\begin{array}{cc} -2.6+i, & |z_{2}\left(t-\tau_{2}\left(t\right)\right)|\leq 1,\\ -2.2+i, & |z_{2}\left(t-\tau_{2}\left(t\right)\right)|>1. \end{array}\right.$$

*Therefore, one can get*

(34) $$\begin{array}{cc} \Phi =\left(\begin{array}{cc} \sqrt{1.48} & \sqrt{4.68}\\ \sqrt{2} & \sqrt{4.68} \end{array}\right) & \Psi =\left(\begin{array}{cc} \sqrt{4.5} & \sqrt{4.25}\\ \sqrt{1.04} & \sqrt{7.76} \end{array}\right) \end{array}$$

*Assume the activation functions of (29) are as follows:*

$$f_{q}\left(z_{q}\right)=0.5{\bar{z}}_{q},\;\;g_{q}\left(z_{q}\right)=\frac{1-e^{-\frac{{\bar{z}}_{q}}{1+|z_{q}|}}}{1+e^{-\frac{{\bar{z}}_{q}}{1+|z_{q}|}}},\;\;q=1,2.$$

*Similarly, one has $L_{f}=L_{g}=\mathrm{diag}\left(0.5,0.5\right)$.*

*We have that*

(35) $$\Lambda -\Phi L_{f}-\Psi L_{g}=\left(\begin{array}{cc} 2.3311 & -2.1124\\ -1.2170 & 2.5255 \end{array}\right)$$

**Figure 3:**
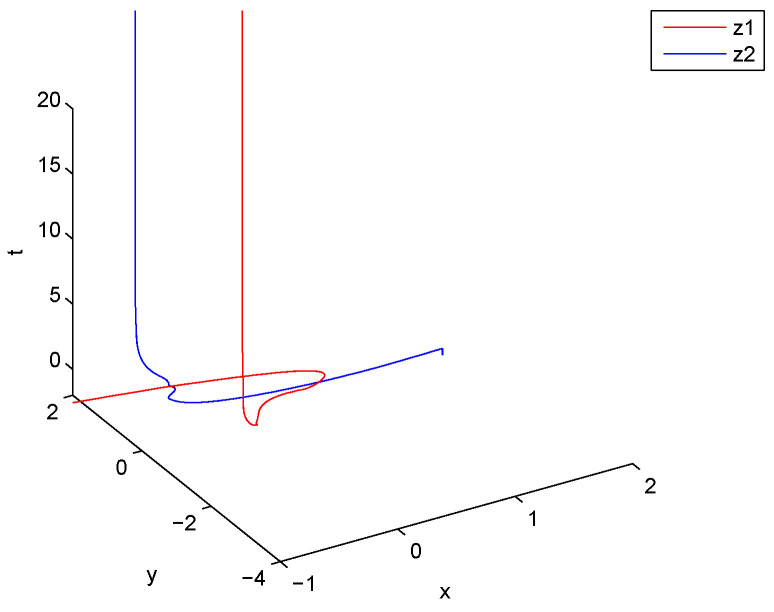
Curves of $z_{1}$ and $z_{2}$.

**Figure 4:**
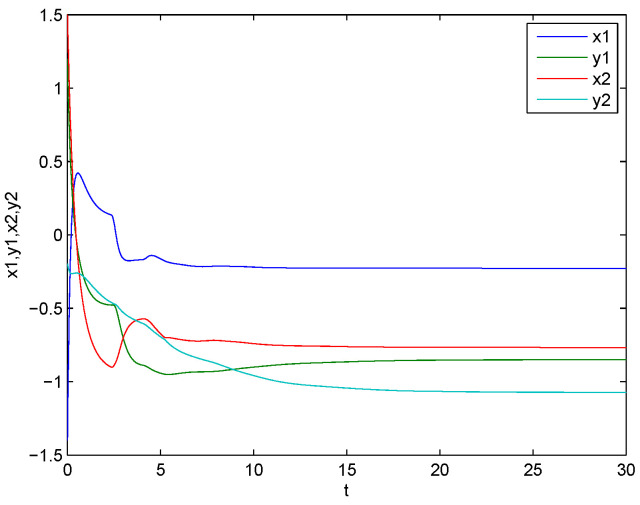
Curves of the real and imaginary parts of $z_{1}$ and $z_{2}$.

*is an M matrix, then the conditions of Theorem 1 are satisfied and the CVMRNN system (29) is global exponentially stable. Numerical simulations are shown in Figure 3 and Figure 4.*

**Remark** **3.** *Examples 1 and 2 show that the constraints on the activation functions are more relaxed. In Example 1, $f_{q}\left(\cdot \right)$ and $g_{q}\left(\cdot \right)$ only need to satisfy the Lipschitz condition, and the partial derivative of $f_{q}\left(\cdot \right)$ and $g_{q}\left(\cdot \right)$ need not be existent, bounded, and continuous, unlike in [3,27]—that is, $\frac{\partial f_{j}^{R}}{\partial x_{j}},\frac{\partial f_{j}^{R}}{\partial y_{j}},\frac{\partial f_{j}^{I}}{\partial x_{j}},\frac{\partial f_{j}^{I}}{\partial y_{j}}$ need not be existent, bounded, and continuous. In Example 2, $f_{q}\left(\cdot \right)$ and $g_{q}\left(\cdot \right)$ need not be explicitly described by dividing their real and imaginary parts, unlike in [3,17,18,25,26,27,28,29,30].*

## 5. Conclusions

In this paper, the existence, uniqueness, and exponential stability of the equilibrium point for a class of MCVNNs with time delays were investigated. Several sufficient conditions were obtained by means of the *M*-matrix theorem and Brouwer’s fixed-point theorem. These results obtained can be applied to general MCVNNs where the activation functions are explicitly described by either dividing the real parts and imaginary parts or not. Two numerical examples were provided, while our corresponding analysis demonstrates that the theoretic results obtained are viable for the design and application of MCVNNs with time delays.
